# Foliar Spray of Micronutrients Alleviates Heat and Moisture Stress in Lentil (*Lens culinaris* Medik) Grown Under Rainfed Field Conditions

**DOI:** 10.3389/fpls.2022.847743

**Published:** 2022-04-07

**Authors:** Visha Kumari Venugopalan, Rajib Nath, Kajal Sengupta, Anjan K. Pal, Saon Banerjee, Purabi Banerjee, Malamal A. Sarath Chandran, Suman Roy, Laxmi Sharma, Akbar Hossain, Kadambot H. M. Siddique

**Affiliations:** ^1^Department of Agronomy, Bidhan Chandra Krishi Viswavidyalaya, Mohanpur, India; ^2^Indian Council of Agricultural Research (ICAR)-Central Research Institute for Dryland Agriculture, Hyderabad, India; ^3^Department of Crop Physiology, Bidhan Chandra Krishi Viswavidyalaya, Mohanpur, India; ^4^Department of Agricultural Meteorology and Physics, Bidhan Chandra Krishi Viswavidyalaya, Mohanpur, India; ^5^Indian Council of Agricultural Research (ICAR)-Central Research Institute for Jute and Allied Fibers, Kolkata, India; ^6^Department of Agronomy, Bangladesh Wheat and Maize Research Institute, Dinajpur, Bangladesh; ^7^The University of Western Australia (UWA), Institute of Agriculture and School of Agriculture and Environment, The University of Western Australia, Perth, WA, Australia

**Keywords:** Heat stress, chlorophyll, relative water content, antioxidant machinery, pollen

## Abstract

The simultaneous occurrence of high temperature and moisture stress during the reproductive stage of lentil (*Lens culinaris* Medik) constrains yield potential by disrupting the plant defense system. We studied the detrimental outcomes of heat and moisture stress on rainfed lentils under residual moisture in a field experiment conducted on clay loam soil (Aeric Haplaquept) in eastern India from 2018 to 2019 and from 2019 to 2020 in winter seasons. Lentil was sown on two dates (November and December) to expose the later sowing to higher temperatures and moisture stress. Foliar sprays of boron (0.2% B), zinc (0.5% Zn), and iron (0.5% Fe) were applied individually or in combination at the pre-flowering and pod development stages. High temperatures increased malondialdehyde (MDA) content due to membrane degradation and reduced leaf chlorophyll content, net photosynthetic rate, stomatal conductance, water potential, and yield (kg ha^–1^). The nutrient treatments affected the growth and physiology of stressed lentil plants. The B+Fe treatment outperformed the other nutrient treatments for both sowing dates, increasing peroxidase (POX) and ascorbate peroxidase (APX) activities, chlorophyll content, net photosynthetic rate, stomatal conductance, relative leaf water content (RLWC), seed filling duration, seed growth rate, and yield per hectare. The B+Fe treatment increased seed yield by 35–38% in late-sown lentils (December). In addition, the micronutrient treatments positively impacted physiological responses under heat and moisture stress with B+Fe and B+Fe+Zn alleviating heat and moisture stress-induced perturbations. Moreover, the exogenous nutrients helped in improving physiochemical attributes, such as chlorophyll content, net photosynthetic rate, stomatal conductance, water potential, seed filling duration, and seed growth rate.

## Introduction

Abiotic stresses, such as elevated temperature and moisture stress, are key limiting factors for crop development and output. Climate change has intensified adverse crop environments, which result in significant economic losses in agricultural and horticultural crops (Beck et al., 2007). High temperatures are frequently associated with less water availability. The increased likelihood of combined drought and heat stress events in the future (IPCC, 2014) highlights the need to explore crop responses to these combined stresses and cost-effective management options.

Lentil is a versatile and profitable pulse crop. It is an excellent source of complex carbohydrates, protein, minerals, vitamins, and dietary fiber for humans, and valuable feed and fodder for livestock (Ninou et al., 2019). In India, lentil is a cool-season food legume crop often planted as a rainfed crop during winter. It is grown on 1.51 million ha, producing 1.56 million tons (1,032 kg ha^–1^) (Directorate of Economics and Statistics, 2020). In India’s vast fallow land, lentils can be cultivated on residual soil moisture after the previous rice harvest without additional irrigation. However, the short winter season and temperature fluctuations inhibit lentil growth and productivity. As with chickpea (Kaushal et al., 2013) and lentils (Sita et al., 2017a; Venugopalan et al., 2021a), cool-season food legumes are adapted to low and mild temperature environments and thus susceptible to heat stress (Sita et al., 2017b; Venugopalan et al., 2021b), particularly, during reproductive growth, significantly reducing seed yields (Sita et al., 2017b; Venugopalan et al., 2021b).

Temperatures exceeding 32/20°C (max/min) during lentil flowering and pod filling can significantly impact seed output and quality (Delahunty et al., 2015; Bourgault et al., 2018; Venugopalan et al., 2021b). Furthermore, in eastern India, long-duration rice production prevents the sowing of lentils as a sole crop. Long-term trend analysis data show that late-sown lentils face heat and moisture stress. The crop can experience initial or late moisture stress due to the hard layer of puddled rice soil that depletes soil moisture. The occurrence of moisture and heat stress during the reproductive stage (terminal stage) is a major concern for lentil yield.

Foliar sprays of micronutrients aid their rapid translocation, reducing plant stress, especially under late-sown conditions. Exogenous nutrition administration could be a useful strategy for reducing the adverse effects of heat (Waraich et al., 2012). Zinc (Zn) is essential during the reproductive phase of crops. Zinc-mediated regulation of water relations confers heat tolerance by maintaining cell water and osmotic potential under stress. Iron (Fe) is required for various plant metabolic reactions and helps during stress (Briat et al., 1995; Rout and Sahoo, 2015). Boron (B) is essential for plant reproduction and growth (Dear and Lipsett, 1987; Dell and Huang, 1997). Under stressful conditions, B enhances stomatal opening and gaseous exchange regulation (Chen et al., 2008).

We hypothesize that these micronutrients (Zn, B, and Fe) ameliorate heat and moisture stress in late-sown lentil crops, given their relevance in protecting crops from a wide range of abiotic stresses. We investigated the impact of micronutrient foliar sprays on photosynthesis, stomatal conductance, and reproduction. We conducted a field experiment to (1) determine the effect of sowing time and foliar sprays on the physiochemical properties and yield of lentils and (2) reveal the role of Zn, B, and Fe in alleviating the impact of high temperature and moisture stress.

## Materials and Methods

### Site Characteristics

The field experiments were conducted during the period from 2018 to 19 and from 2019 to 20 in winter seasons at the Seed Farm of Bidhan Chandra Krishi Viswavidyalaya (22°58′ N and 88°32′ E; 9.75 m asl) in Kalyani, West Bengal, India. The study site is flat with a well-drained Gangetic alluvial soil (order: Inceptisol), belonging to the clayey loams, with medium fertility and almost neutral reaction. The soil is low in organic carbon (wet-digestion method), available nitrogen (alkaline permanganate-oxidizable), zinc [diethylenetriaminepentaacetic acid (DTPA)-extractable], boron (azomethine-H), and iron (DTPA-extractable) (0.52%, 138 kg ha^–1^, 0.40 mg kg^–1^, 0.49 mg kg^–1^, and 0.45 mg kg^–1^, respectively), and fairly rich in available P (Brays’ P) and K (NH_4_OAC-extractable) (13 and 132 kg ha^–1^, respectively).

### Treatment Description and Experimental Design

The experiments had a split-plot design with three replications. The main plots were two planting dates: November (normal) and December (late), and the subplots comprised foliar sprays of various micronutrients (see Table 1 for treatment abbreviations and details). A popular red lentil type, *Moitree* (WBL 77), was used due to its medium duration and high production. The foliar sprays were applied during the flowering and pod development stages.

**TABLE 1:** Treatment description and abbreviation.

Treatment	Abbreviation
No spray (Control)	Control
Foliar spray: tap water	Tap water
Foliar spray: 0.5% Zn (ZnSO_4_.7H_2_O)	Zn@0.5%
Foliar spray: 0.5% Fe (FeSO_4_.7H_2_O)	Fe@0.5%
Foliar spray: 0.2% (Na_2_B_4_O_7_.10H_2_O)	B@0.2%
Foliar spray: 0.5% Zn + 0.2% B	Zn+B
Foliar spray: 0.5% Zn + 0.5% Fe	Zn+Fe
Foliar spray: 0.2% B + 0.5% Fe	B+Fe
Foliar spray: 0.5% Zn + 0.5% Fe + 0.2% B	Zn+B+Fe

### Crop Management

Seeds were sown in a 5 × 4 m experimental plot at 30 cm row spacing. Standard crop management procedures were used that include a uniform N: P: K fertilizer dose of 20: 17.5: 334 kg ha^–1^ and one hand weeding 25–30 days after sowing. No irrigation was provided because lentil is grown on residual soil moisture from rainfall during the winter season.

Meteorological data were collected from the All India Coordinated Research Project on Agrometeorology unit, Directorate of Research, Kalyani, West Bengal, from November 2018 to March 2019 and from November 2019 to March 2020. Phenological stage mean rainfall and maximum and minimum temperatures are shown in Table 2. The temperature and moisture status of the soil at various stages of crop growth in both years are given in Supplementary Tables 1, 2.

**TABLE 2:** Mean rainfall and maximum and minimum temperatures during crop phenological stages.

Parameter	Year	Sowing time	Germination	Flowering	Pod initiation	Maturity
Rainfall (mm)	2018–19	Normal	0	21.4	0	0
		Late	5.2	13.6	0.6	51
	2019–20	Normal	0	15.4	0	0
		Late	0	0	0	0.3
Tmax (°C)	2018–19	Normal	29.6	26.4	24.7	25.9
		Late	25.7	24.3	26.5	29.4
	2019–20	Normal	30.1	25.5	23.1	26.4
		Late	27	22.4	24	29.8
Tmin (°C)	2018–19	Normal	17.7	13.6	9.2	10.7
		Late	10.5	9.6	11.9	11.5
	2019–20	Normal	18.1	13.6	11.5	12.8
		Late	15.4	11	11.1	16.7

### Measurement of Physiological Parameters

#### Chlorophyll Content

The chlorophyll content in leaf samples was estimated as per Arnon (1949). Absorbance was read at 480, 510, 645, and 663 nm using a Systronics-105 spectrophotometer against a blank containing 80% acetone. The amount of chlorophyll *a*, chlorophyll *b*, total chlorophyll, and carotenoids was estimated as follows:


$$\begin{array}{c} \mathrm{mg of chlorophyll}\;a\;{\text{g}}^{-1}\;\mathrm{of fresh weight}\\ \;=\left[\left(12.7\times A_{663}\right)-\left(2.69\times A_{645}\right)\right]\times \left(\frac{V}{W}\times 1,000\right) \end{array}$$

$$\begin{array}{c} \mathrm{mg of chlorophyll}\text{ b }{\text{g}}^{-1}\;\mathrm{of fresh weight}\\ \;=\left[\left(22.9\times A_{665}\right)-\left(4.68\times A_{663}\right)\right]\times \left(\frac{V}{W}\times 1,000\right) \end{array}$$

$$\begin{array}{c} \mathrm{mg of total chlorophyll}\;{\text{g}}^{-1}\;\mathrm{of fresh weigh}\\ \;=\left[\left(12.7\times A_{663}\right)-\left(2.69\times A_{645}\right)\right]\times \left(\frac{V}{W}\times 1,000\right) \end{array}$$

$$\begin{array}{c} \mathrm{mg of carotenoid}\;{\text{g}}^{-1}\;\mathrm{of fresh weight}\\ \;=\left[\left(7.6\times A_{480}\right)-\left(1.49\times A_{510}\right)\right]\times \left(\frac{V}{W}\times 1,000\right) \end{array}$$

where V = volume of extract (ml), W = fresh weight (FW) of tissue (g), and A = absorbance.

#### Pollen Studies

Pollen viability was estimated by acetocarmine stain (Srinivasan and Gaur, 2011) and expressed in percentage. Pollen fertility was determined by their staining ability. Pollen germination was estimated as per Niles and Quesenberry (1992). Pollen germination was recorded at 30-min intervals for up to 90 min, by which time the pollen had reached maximum germination percentage. The slides were viewed under a microscope (10×).

#### Seed Growth Rate and Seed Filling Duration

Five pods per plant (three plants from each replicate; nine plants total) were tagged at the beginning of pod filling (pod size ≈1 cm) and followed to physiological maturity to investigate seed growth rate and seed-filling duration, as reported by Sehgal et al. (2019). Seed dry weight was recorded 7 days after pod filling started and at physiological maturity after oven-drying at 45°C for 5 days. The time (days) taken for tagged pods to complete seed filling was noted.

#### Relative Leaf Water Content

Relative leaf water content (RLWC) was estimated as per Perez et al. (2002). Leaves (250 mg) were collected from five plants in each replication, cut into small pieces, and pooled to record FW. The leaves were immersed in double-distilled water for 4 h, before recording turgid weight (TW), and then dried at 80 ± 1°C in a hot air oven to constant weight to record dry weight (DW). The RLWC was expressed as follows:


$$RLWC=\frac{FW-DW}{TW-DW}\times 100$$

#### Net Photosynthetic Rate, Transpiration Rate, and Stomatal Conductance

Leaf net photosynthetic rate (Pn), transpiration rate (E), and stomatal conductance (C) were measured using a portable handheld photosynthesis system (CI-340 Handheld Photosynthesis system, CID Bio-Science, Inc. Camas, WA 98607, United States). The measurements were taken from fully developed upper leaves of three selected plants on clear sunny days between 10.30 a.m. and 12.30 p.m. at the 100% pod development stage.

#### Enzyme Assays

Ascorbate peroxidase (APX) and peroxidase (POX) activities were measured according to the protocols by Nakano and Asada (1981) and Castillo et al. (1984), respectively. To extract the APX enzyme, a 1 g leaf sample was frozen in liquid nitrogen to suppress proteolytic activity, then ground in 10 ml of extraction buffer [0.1 M phosphate buffer, pH 7.5, with 0.5 mM ethylenediaminetetraacetic acid (EDTA)], passed through four layers of cheesecloth, and centrifuged at 15,000 *g* for 20 min. The absorbance of the supernatant was read at 290 nm at 1-min intervals for 5–10 min. The absorbance coefficient of ascorbic acid was 2.8 mM^–1^ cm^–1^. The POX extract was prepared by freezing a 1 g leaf sample in liquid nitrogen to prevent proteolytic activity, then grinding with 10 ml of extraction buffer (0.1 M phosphate buffer pH 7.5, containing 0.5 mM EDTA). The absorbance due to the formation of tetra-guaiacol was recorded at 470 nm, and the enzyme activity was calculated as the extinction coefficient of its oxidation product, tetra-guaiacol ε = 26.2 mM^–1^ cm^–1^.

#### Lipid Peroxidation

Lipid peroxidation is the oxidative degradation of lipid-fatty acid by reactive oxygen species (ROS). The level of lipid peroxidation is measured in terms of thiobarbituric acid (TBA) reactive substances content (Heath and Packer, 1968) and expressed as malondialdehyde (MDA). A total of 4 ml of 0.5% TBA in 20% trichloroacetic acid (TCA) was added to a 1.0 ml aliquot of the supernatant, heated at 95°C for 30 min in a laboratory water bath, and cooled in an ice bath. After cooling, the aliquot was centrifuged at 10,000 *g* for 10 min. The absorbance of the clear supernatant was recorded at 532 nm. Values of non-specific absorption recorded at 600 nm were subtracted from the values recorded at 532 nm. The MDA content is calculated according to its extinction coefficient ε = 155 mM cm^–1^.

#### Proline

Free proline content in the leaves was determined using the method of Bates et al. (1973).

#### Grain Micronutrient Concentration

Zinc and Fe were analyzed using an atomic absorption spectrometer (210/211 VGP, United States). Boron was estimated according to the azomethine-H method (Lohse, 1982).

#### Protein Analysis

The finely ground seed sample (0.5 g) was digested with concentrated H_2_SO_4_. Total nitrogen in seeds was determined by the micro-Kjeldahl method as per the procedure suggested by Baethgen and Alley (1989). Seed protein contents were calculated by multiplying the nitrogen values by 6.25. Triplicate analyses were carried out on each sample.

#### Soil Moisture Estimation

Soil moisture measurement was carried out gravimetrically. Moisture was recorded at three depths: 0–15 cm, 15–30 cm, and 30–45 cm. All samples were dried in an oven at 105°C for 24–48 h to constant moisture. The dried soil samples were weighed on an electrical balance. Actual moisture content in each soil sample was calculated as follows:


$$\begin{array}{c} \mathrm{Soil moisture}\;(\%)\\ =\frac{Freshweightofsoil\left(g\right)-Dryweightofsoil\left(g\right)}{Dryweightofsoil\left(g\right)}\times 100 \end{array}$$

From percent soil moisture, soil moisture on depth basis was estimated using:


$$\mathrm{Soil}\mathrm{moisture}\left(\text{cm}\right)=\mathrm{Soil}\mathrm{moisture}\mathrm{content}\left(\%\right)\times {\text{A}}_{\text{i}}\times \text{D}$$

#### Flower and Pod Numbers and Yield

The number of open flowers per plant was recorded daily, excluding the previous day’s open flowers in the following day’s count. Five plants were tagged in the central rows from each plot to calculate the total flower number. Five plants were randomly uprooted from the sampling rows of each plot at harvest to calculate the average pod number. The grain from each plot was dried to 12–13% moisture to calculate seed yield per hectare.

### Statistical Analysis

The data were analyzed using analysis of variance (ANOVA) for split-plot design (Gomez and Gomez, 1984). IRRI’s STAR software was used to perform the statistical analysis. Tukey’s *post-hoc* test was applied to compare differences between the mean values.

## Results

### Chlorophyll Content

**FIGURE 1:**
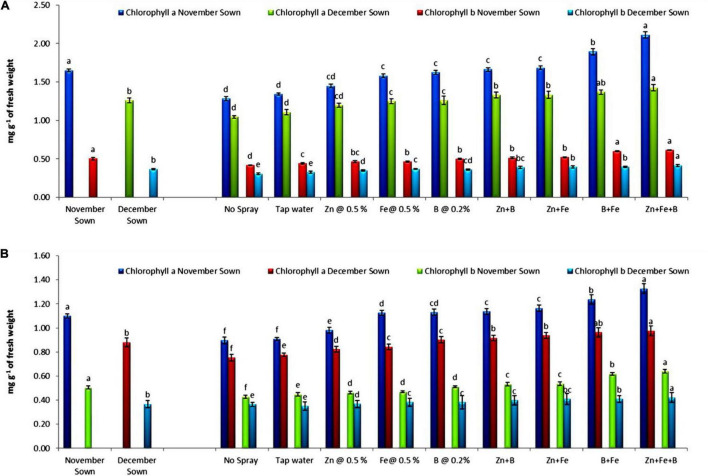
Leaf chlorophyll a and b for different sowing dates and foliar spray treatments in the **(A)** 2018–19 and **(B)** 2019–20 lentil cropping seasons. Values are means, and bars represent ± SEM (*n* = 3). Different letters indicate significant differences between means.

**FIGURE 2:**
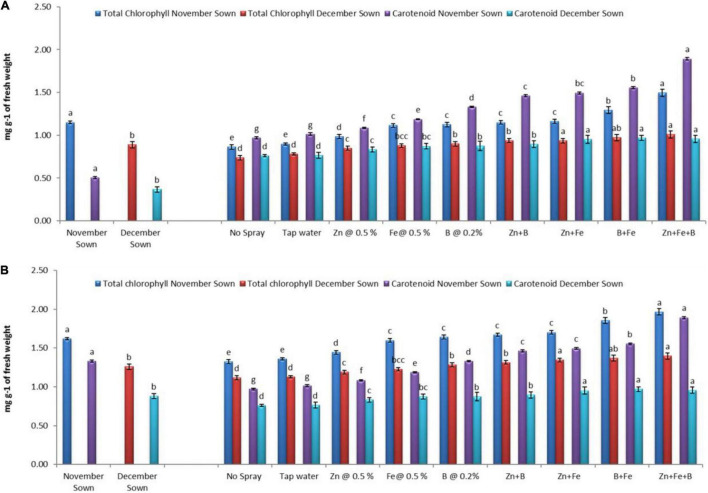
Total chlorophyll content and carotenoid for different sowing dates and foliar spray treatments in the **(A)** 2018–19 and **(B)** 2019–20 lentil cropping seasons. Values are means, and bars represent ± SEM (*n* = 3). Different letters indicate significant differences between means.

Normal-sown (November) lentils had higher values of chlorophyll a, chlorophyll b, total chlorophyll, and carotenoid (1.15, 0.50, 1.65, and 1.36 mg g^–1^ FW) than late-sown (December) lentils (0.89, 0.37, 1.26, and 0.88 mg g^–1^ FW) in 2018–19 (Figures 1, 2), with a similar trend in 2019–20. For normal-sown lentils in 2018–19, the micronutrient foliar sprays increased chlorophyll contents. The Zn+Fe+B treatment recorded the highest values for chlorophyll a, chlorophyll b, total chlorophyll, and carotenoid (1.48, 0.62, 2.11, and 1.89 mg g^–1^) relative to the control (0.86, 0.42, 1.28, and 0.97 mg g^–1^). Late-sown lentils had lower values than normal-sown lentils due to moisture and temperature stress, but the foliar sprays somewhat ameliorated the stress effects with higher values than the control. A similar trend occurred in 2019–20 (Figures 1, 2).

### Pollen Studies

**FIGURE 3:**
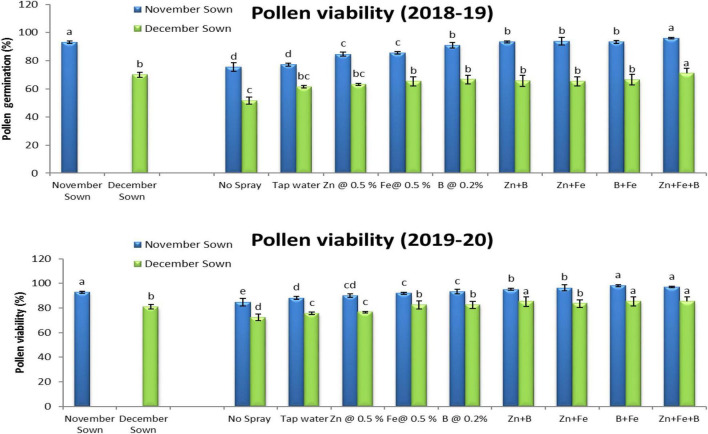
Pollen viability (%) for different sowing dates and foliar spray treatments in the 2018–19 and 2019–20 lentil cropping seasons. Values are means, and bars represent ± SEM (*n* = 3). Different letters indicate significant differences between means.

**FIGURE 4:**
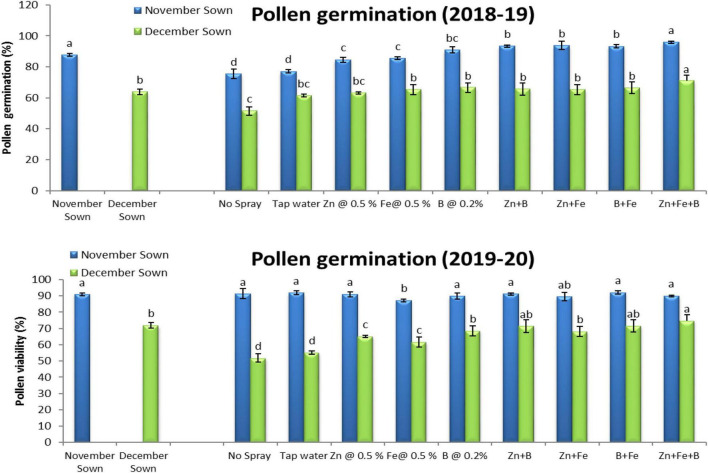
Pollen germination (%) for different sowing dates and foliar spray treatments in the 2018–19 and 2019–20 lentil cropping seasons. Values are means, and bars represent ± SEM (*n* = 3). Different letters indicate significant differences between means.

Normal-sown lentils had 92% pollen viability and 88% pollen germination in 2018–19 as compared to 70 and 64%, respectively, for late-sown lentils (Figures 3, 4). A similar trend occurred in 2019–20. The Zn+Fe+B and Fe+B foliar sprays produced the highest pollen viability and germination relative to the control (no spray) (Supplementary Figure 1).

### Seed Growth Rate and Seed Filling Duration

Normal-sown lentils had a higher seed growth rate (7.69 and 7.75 g DW) than late-sown lentils (4.52 and 4.53 g DW) in 2018–19 and 2019–20, respectively. Stress reduced the days to maturity, resulting in a lower seed growth rate in the late-sown crop (Table 3). The B+Fe treatment had 25 and 14% higher seed growth rates in 2018–19 and 2019–20 than the control.

**TABLE 3:** Effect of sowing time and zinc, iron, and boron foliar sprays on seed growth rate and seed filling duration of lentil.

Treatments	Seed growth rate (g dry weight) (2018–19)		Seed growth rate (g dry weight) (2019–20)		Seed filling duration (days) (2018–19)		Seed filling duration (days) (2019–20)	
	Normal-sown	Late-sown	Normal-sown	Late-sown	Normal-sown	Late-sown	Normal-sown	Late-sown
Control	6.53 ± 0.05^c^	4.10 ± 0.30^e^	7.23 ± 0.15^c^	4.10 ± 0.12^c^	18.00 ± 0.24^c^	12.33 ± 0.01^c^	18.67 ± 0.21^c^	13.00 ± 0.02^d^
Tap water	6.80 ± 0.20^c^	4.17 ± 0.3^e^	7.33 ± 0.24^c^	4.20 ± 0.14^c^	18.00 ± 0.15^c^	12.00 ± 0.01^c^	18.67 ± 0.21^c^	13.33 ± 0.01^c^
Zn@0.5%	7.57 ± 0.40^b^	4.30 ± 0.50^d^	7.90 ± 0.31^b^	4.30 ± 0.12^c^	19.00 ± 0.12^b^	12.33 ± 0.01^c^	19.33 ± 0.34^b^	13.33 ± 0.31^c^
Fe@0.5%	8.07 ± 0.40*ab*	4.37 ± 0.40^d^	8.00 ± 0.30^b^	4.43 ± 0.14^b^	19.00 ± 0.31^b^	12.67 ± 0.02^b^	19.33 ± 0.11^b^	13.33 ± 0.21^c^
B@0.2%	8.20 ± 0.45^a^	4.2*d*3 ± 0.3*d*	8.20 ± 0.21*ab*	4.52 ± 0.22^b^	19.67 ± 0.02^a^	12.67 ± 0.57^b^	20.00 ± 0.25^a^	13.67 ± 0.24^b^
Zn+B	7.93 ± 0.05^b^	4.53 ± 0.05^c^	7.80 ± 0.24^b^	4.53 ± 0.20^b^	19.67 ± 0.14^a^	12.67 ± 0.57^b^	20.33 ± 0.32^a^	13.67 ± 0.21^b^
Zn+Fe	7.53 ± 0.20^b^	4.70 ± 0.36^b^	7.53 ± 0.24^c^	4.60 ± 0.31^b^	19.67 ± 0.12^a^	12.67 ± 0.54^b^	19.67 ± 0.32^b^	13.67 ± 0.12^b^
B+Fe	8.37 ± 0.66^a^	4.93 ± 0.15^a^	8.40 ± 0.25^a^	4.93 ± 0.25^a^	19.67 ± 0.12^a^	14.00 ± 0.45^a^	20.67 ± 0.14^a^	15.00 ± 0.14^a^
Zn+B+Fe	8.20 ± 0.62^a^	4.83 ± 0.30^a^	8.33 ± 0.24^a^	4.63 ± 0.47^b^	19.33 ± 0.14^b^	14.00 ± 0.48^a^	20.33 ± 0.24^a^	15.00 ± 0.14^a^

*Values are means, and numbers with parentheses are ± SEM (n = 3). Different letters indicate significant differences between means.*

### Relative Leaf Water Content

Relative leaf water content significantly differed between sowing dates and foliar sprays (Table 4). The late-sown crop had 23–24% lower RLWC than the normal sown crop in both years. Foliar spray of Zn+Fe, B+Fe, and Zn+B+Fe increased RLWC in both the years relative to the control.

**TABLE 4:** Effect of sowing time and zinc, iron, and boron foliar sprays on the relative leaf water content of lentil (%).

Treatments	2018–19		2019–20	
	Normal-sown	Late-sown	Normal-sown	Late-sown
Control	79.60 ± 1.62^c^	60.83 ± 0.72^c^	79.07 ± 0.99^c^	60.03 ± 0.73^c^
Tap water	80.53 ± 1.46*bc*	60.93 ± 0.68^c^	80.58 ± 0.55*bc*	60.68 ± 1.05^c^
Zn@0.5%	80.60 ± 1.03^b^	61.67 ± 1.03^b^	80.80 ± 0.48^b^	60.72 ± 0.74*bc*
Fe@0.5%	80.73 ± 0.40^b^	61.33 ± 1.25^b^	80.67 ± 0.58^b^	60.53 ± 1.08^c^
B@0.2%	80.67 ± 0.99^b^	61.08 ± 1.47^b^	81.62 ± 0.60^b^	61.43 ± 0.92^b^
Zn+B	81.83 ± 0.29^a^	61.77 ± 0.28	81.78 ± 0.52^b^	61.72 ± 1.29^b^
Zn+Fe	81.83 ± 1.53^a^	62.60 ± 1.49^a^	81.52 ± 0.38^b^	61.70 ± 0.96^b^
B+Fe	81.60 ± 0.55^a^	62.60 ± 1.57^a^	82.22 ± 0.54^a^	62.05 ± 1.08^a^
Zn+B+Fe	81.82 ± 0.78^a^	62.23 ± 1.32^a^	82.20 ± 0.79^a^	62.32 ± 0.25^a^

*Values are means, and numbers with parentheses are ± SEM (n = 3). Different letters indicate significant differences between means.*

### Net Photosynthetic Rate, Transpiration Rate, and Stomatal Conductance

**FIGURE 5:**
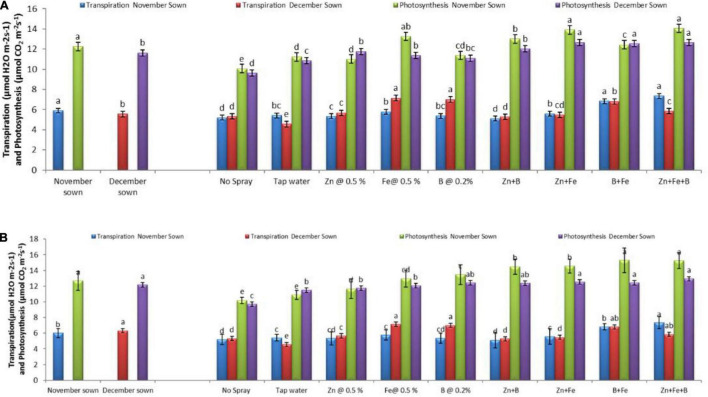
Transpiration and photosynthesis (μmol CO_2_ m^– 2^ s^– 1^) for different sowing dates and foliar spray treatments in the **(A)** 2018–19 and **(B)** 2019–20 lentil cropping seasons. Values are means, and bars represent ± SEM (*n* = 3). Different letters indicate significant differences between means.

**FIGURE 6:**
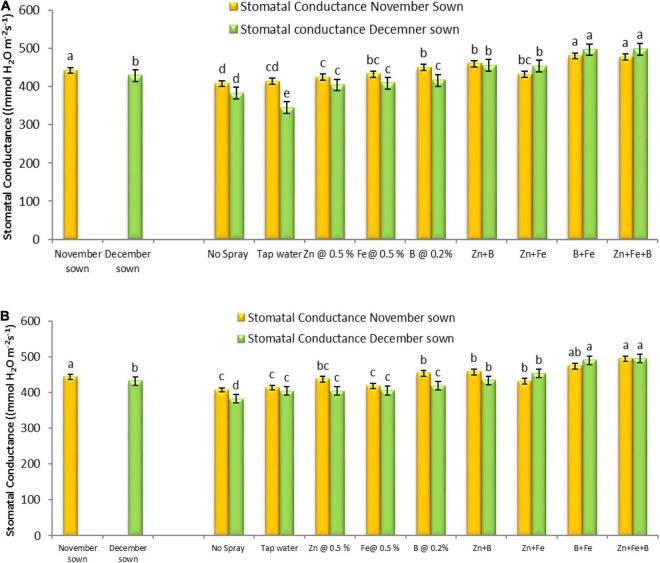
Stomatal conductance (μmol CO_2_ m^– 2^ s^– 1^) for different sowing dates and foliar spray treatments in the **(A)** 2018–19 and **(B)** 2019–20 lentil cropping seasons. Values are means, and bars represent ± SEM (*n* = 3). Different letters indicate significant differences between means.

Normal-sown lentils had higher maximum net photosynthesis (12.26 and 12.68 μmol CO_2_ m^–2^ s^–1^), transpiration rate (5.90 and 6.01 μmol H_2_O m^–2^ s^–1^), and stomatal conductance (441.59 and 442.70 mmol H_2_O m^–2^ s^–1^) than late-sown lentils (11.62 and 12.15 μmol CO_2_ m^–2^ s^–1^, 5.55 and 6.32 μmol H_2_O m^–2^ s^–1^, and 428.65 and 431.63 mmol H_2_O m^–2^ s^–1^) in the period of 2018–19 and 2019–20, respectively. The foliar micronutrient sprays also affected net photosynthetic rate, transpiration rate, and stomatal conductance (Figures 5, 6).

### Enzymes

**FIGURE 7:**
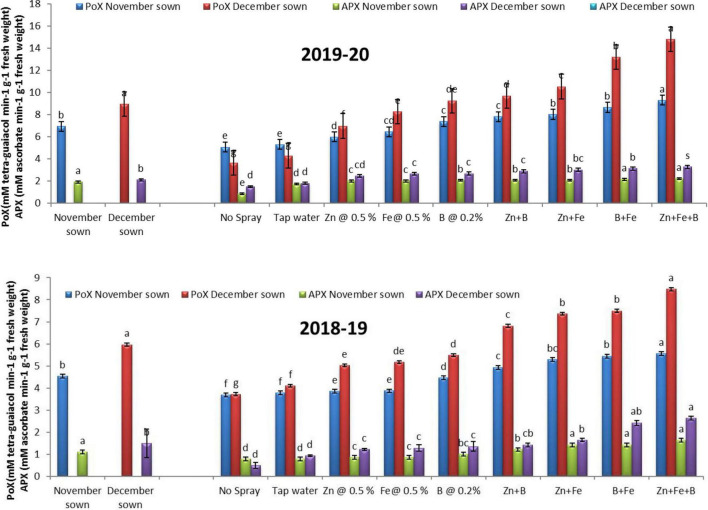
Peroxidase (POX; mM tetra-guaiacol min^– 1^ g^– 1^ FW) and ascorbate peroxidase (APX; mM ascorbate min^– 1^ g^– 1^ FW) activities for different sowing dates and foliar spray treatments in 2018–19 and 2019–20 lentil cropping seasons. Values are means, and bars represent ± SEM (*n* = 3). Different letters indicate significant differences between means.

Late-sown lentils had higher POX (6.92 and 8.94 mM tetra-guaiacol min^–1^ g^–1^ FW) and APX (1.90 and 2.12 mM ascorbate min^–1^ g^–1^ FW) activities than normal-sown lentils (4.54 and 7.09 mM tetra-guaiacol min^–1^ g^–1^ FW and 1.34 and 1.48 mM ascorbate min^–1^ g^–1^ FW) in 2018–19 and 2019–20, respectively (Figure 7).

### Lipid Peroxidation

**FIGURE 8:**
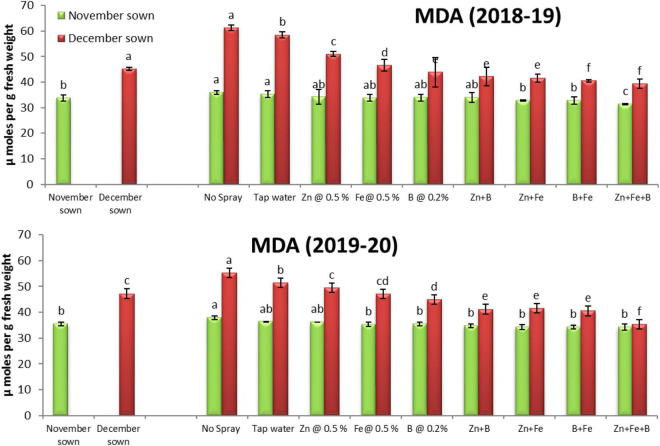
Malondialdehyde (MDA; μmol g^– 1^ FW) content for different sowing dates and foliar spray treatments in the 2018–19 and 2019–20 lentil cropping seasons. Values are means, and bars represent ± SEM (*n* = 3). Different letters indicate significant differences between means.

Late-sown lentils had significantly higher lipid peroxidation than normal-sown lentils. The foliar spray treatments did not significantly affect MDA contents in normal-sown lentils but significantly increased them in late-sown lentils (Figure 8).

### Proline

**FIGURE 9:**
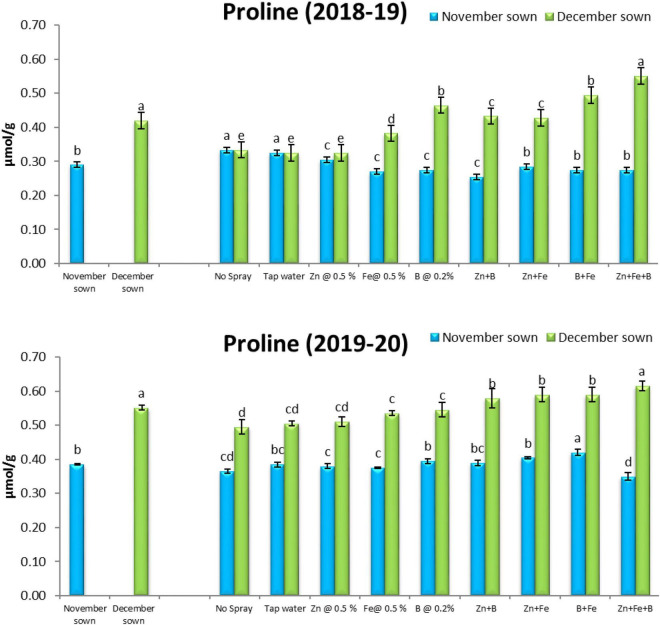
Proline (μmol g^– 1^) content for different sowing dates and foliar spray treatments in the 2018–19 and 2019–20 lentil cropping seasons. Values are means, and bars represent ± SEM (n = 3). Different letters indicate significant differences between means.

Late-sown lentils accumulated more proline (0.40 and 0.52 μmol g^–1^) than normal-sown lentils (0.29 and 0.39 μmol g^–1^) in 2018–19 and 2019–20, respectively (Figure 9).

### Moisture Storage

**FIGURE 10:**
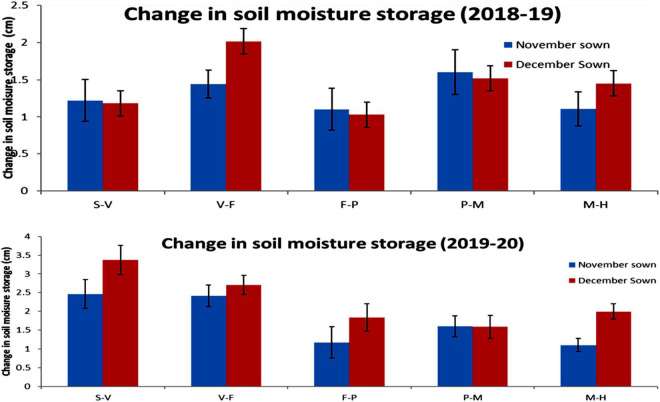
Change in soil moisture storage (0–45 cm soil profile) for different sowing dates in the 2018–19 and 2019–20 lentil cropping seasons. Error bars represent the standard error of the mean. Values are means, and bars represent ± SEM (*n* = 3). Different letters indicate significant differences between means. S–V (sowing to the vegetative stage), V–F (vegetative to the flowering stage), F–P (flowering to pod development stage), P–M (pod development to maturity stage), and M–H (maturity to harvest stage).

Late-sown lentils had lower soil moisture content than normal-sown lentils throughout both growing seasons, except initially in the second year due to a fair amount of rain (15.4 mm) just before sowing. Changes in moisture storage over time are shown in Figure 10.

### Seed Nutrient and Protein Contents

The Fe, Zn, and B contents did not significantly differ between growing seasons, so the data were pooled (Table 5). Late sowing significantly affected micronutrient accumulation and seed protein content. Late-sown lentils had 21.85–23.13% seed protein when compared with 24.32–25.05% in normal-sown lentils. Foliar sprays at the pre-flowering and pod development stages also significantly affected micronutrient accumulation.

**TABLE 5:** Effect of sowing time and zinc, iron, and boron foliar sprays seed nutrients and seed protein.

Treatments	Fe (mg/100 g)		Zn (mg/100 g)		B (mg/100 g)		Seed protein (%)	
	Normal-sown	Late-sown	Normal-sown	Late-sown	Normal-sown	Late-sown	Normal-sown	Late-sown
Control	7.28 ± 0.2^b^	3.52 ± 0.2^b^	3.13 ± 0.3^b^	3.13 ± 0.2^b^	3.52 ± 0.2^b^	1.40 ± 0.2^d^	24.32 ± 0.3^b^	21.85 ± 0.4^c^
Tap water	7.32 ± 0.1^b^	3.50 ± 0.2^b^	3.25 ± 0.3^b^	3.25 ± 0.2^b^	3.50 ± 0.2^b^	1.47 ± 0.2^d^	24.58 ± 0.1*ab*	21.98 ± 0.2^c^
Zn@0.5%	7.33 ± 0.3^b^	4.13 ± 0.1^a^	3.35 ± 0.1^b^	3.35 ± 0.5^b^	4.13 ± 0.2^a^	1.90 ± 0.1^a^	24.90 ± 0.1^a^	22.17 ± 0.5^b^
Fe@0.5%	7.80 ± 0.2^a^	3.72 ± 0.2^b^	3.37 ± 0.2^b^	3.37 ± 0.2^b^	3.72 ± 0.6^b^	1.72±*c*0.2	24.80 ± 0.2^a^	22.35 ± 0.6^b^
B@0.2%	7.52 ± 0.3^b^	3.67 ± 0.3^b^	4.05 ± 0.2^a^	4.05 ± 0.1^a^	3.67 ± 0.2^b^	1.73 ± 0.3^c^	24.80 ± 0.2^a^	22.43 ± 0.1^b^
Zn+B	7.58 ± 0.2^b^	4.22 ± 0.4^a^	3.92±*a*0.1*b*	3.92 ± 0.1*ab*	4.22 ± 0.1^a^	1.83 ± 0.1^b^	24.90 ± 0.3^a^	22.87 ± 0.3^b^
Zn+Fe	7.88 ± 0.3^a^	4.33 ± 0.2^a^	3.48 ± 0.2^b^	3.48 ± 0.4^b^	4.33 ± 0.2^a^	1.95 ± 0.2^a^	24.98 ± 0.1^a^	22.82 ± 0.2^b^
B+Fe	7.78 ± 0.1^a^	3.78 ± 0.1^b^	4.02 ± 0.2^a^	4.02 ± 0.2^a^	3.78 ± 0.2^b^	1.90 ± 0.2^a^	25.03 ± 0.2^a^	22.90 ± 0.3^a^
Zn+B+Fe	7.77 ± 0.2^a^	4.18 ± 0.2^a^	4.00 ± 0.2^a^	4.00 ± 0.1^a^	4.18 ± 0.2^a^	1.92 ± 0.1^a^	25.05 ± 0.1^a^	23.12 ± 0.1^a^

*Values are means, and numbers with parentheses are ± SEM (n = 3). Different letters indicate significant differences between means.*

### Flowers Pods and Yield

Sowing date significantly affected pod number per plant in both growing seasons. Foliar spray of micronutrients at pre-flowering and pod development stages increased pod number per plant by 75–104.67 and 87.33–138.83 in 2018–19 and 2019–20, respectively, relative to the control (Table 6). Lentil seed yield significantly differed between treatments in both years (Table 7).

**TABLE 6:** Effect of sowing time and zinc, iron, and boron foliar sprays on flower and pod numbers.

Treatments	2018–19				2019–20			
	Flower number		Pod number		Flower number		Pod number	
	Normal-sown	Late-sown	Normal-sown	Late-sown	Normal-sown	Late-sown	Normal-sown	Late-sown
Control	275 ± 8.4^c^	204 ± 12.1^c^	83 ± 6.5^c^	68 ± 6.5^c^	288 ± 7.3^d^	218 ± 13.2^c^	92 ± 8.7^e^	83 ± 7.7^b^
Tap water	282 ± 8.7^c^	224 ± 11.2^c^	90 ± 6.4^c^	68 ± 3.5^c^	285 ± 4.5^d^	234 ± 9.2*bc*	104 ± 5.1^d^	85 ± 4.5^b^
Zn@0.5%	306 ± 5.6^b^	256 ± 10.3^b^	109 ± 3.5*ab*	71 ± 6.7^b^	313 ± 5.7^b^	256 ± 8.4^b^	134 ± 6.2^c^	87 ± 1.5^b^
Fe@0.5%	299 ± 2.3^c^	264 ± 9.5^a^	106 ± 2.7*ab*	81 ± 7.6^a^	307 ± 7.7^c^	268 ± 10.2^a^	144 ± 3.5^c^	90 ± 2.5^a^
B@0.2%	318 ± 6.4^b^	271 ± 6.8^a^	104 ± 7.8*ab*	79 ± 8.4*ab*	318 ± 15.1^b^	275 ± 5.4^a^	150 ± 4.1^b^	88 ± 4.8^b^
Zn+B	307 ± 11.2^b^	275 ± 8.9^a^	116 ± 8.4^a^	84 ± 6.5^a^	319 ± 5.7^b^	275 ± 6.5^a^	159 ± 9.5^b^	94 ± 9.5^a^
Zn+Fe	304 ± 12.5*bc*	249 ± 9.7^b^	129 ± 9.4^a^	73 ± 8.4*ab*	305 ± 6.1^c^	275 ± 11.2^a^	157 ± 7.4^b^	97 ± 6.5^a^
B+Fe	332 ± 9.8^a^	262 ± 9.7^a^	119 ± 8.4^a^	75 ± 7.8*ab*	341 ± 9.5^a^	269 ± 6.4^a^	179 ± 8.4^a^	99 ± 6.7^a^
Zn+B+Fe	357 ± 8.6^a^	289 ± 5.6^a^	101 ± 5.5*ab*	84 ± 9.7^a^	357 ± 6.2^a^	291 ± 7.2^a^	172 ± 8.7^a^	95 ± 5.8^a^

*Values are means, and numbers with parentheses are ± SEM (n = 3). Different letters indicate significant differences between means.*

**TABLE 7:** Effect of sowing time and zinc, iron, and boron foliar sprays on seed yield (kg ha^–1^).

Treatments	2018–19		2019–20	
	Normal-sown	Late-sown	Normal-sown	Late-sown
Control	1,024 ± 24.9*e*	861 ± 35.2^e^	1,011 ± 29.5*f*	865 ± 35.2^d^
Tap water	1,033 ± 31.2*e*	887 ± 44.6^e^	1,033 ± 60.1*f*	876 ± 63.2^d^
Zn@0.5%	1,264 ± 22.0*d*	905 ± 28.5^d^	1,288 ± 38.5*d*	919 ± 54.4^c^
Fe@0.5%	1,194 ± 35.2*d*	924 ± 54.2^d^	1,179 ± 53.2*e*	954 ± 29.5^c^
B@0.2%	1,325 ± 46.2*c*	957 ± 24.5*cd*	1,363 ± 46.2*c*	961 ± 36.5*bc*
Zn+B	1,483 ± 32.8*b*	973 ± 36.4^c^	1,496 ± 57.5*c*	965 ± 54.2^b^
Zn+Fe	1,481 ± 19.7*b*	992 ± 29.5^c^	1,492 ± 46.5*c*	996 ± 44.6^b^
B+Fe	1,744 ± 38.1*a*	1,183 ± 32.7*a*	1,723 ± 54.9*a*	1,073 ± 52.1*a*
Zn+B+Fe	1,525 ± 55.8*b*	1,064 ± 58.4*b*	1,534 ± 81.4*b*	1,004 ± 36.4*a*

*Values are means, and numbers with parentheses are ± SEM (n = 3). Different letters indicate significant differences between means.*

### Correlation Studies

**FIGURE 11:**
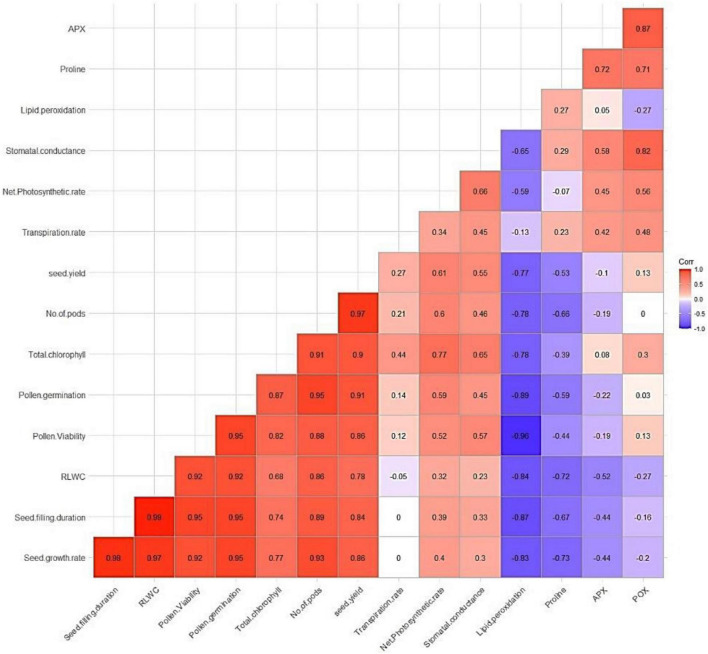
Correlation analysis of physiological traits and yield.

Pooled data for both years were used for the correlation analysis. The correlation coefficients for different crop parameters and grain yield indicated that most of the agro-physiological crop parameters were—flower number, pod number, seed growth rate, seed filling duration, total chlorophyll, and enzyme activities—positively correlated with yield, while lipid peroxidation and proline content were negatively correlated with growth and yield parameters (Figure 11).

The correlation matrix heatmap shows the Pearson’s correlation coefficient values for all studied parameters, with positive values in red and negative values in blue. The values range from −1 to 1, where −1 indicates a perfect negative linear relationship, 1 indicates a perfect positive linear relationship, and 0 indicates no relationship between variables. The studied parameters were seed growth rate, seed filling duration, RLWC, pollen viability, pollen germination, total chlorophyll, pod number, seed yield, transpiration rate, net photosynthetic rate, stomatal conductance, lipid peroxidation, proline, and APX (color figure online).

## Discussion

### Relative Water Content to Water Relations and Gas Exchange

In terms of the physiological consequences of cellular water shortage, the relative amount of water present in plant tissues is the most accurate indicator of plant water status (Barrs and Weatherly, 1962; Nilsen and Orcutt, 1996). Late-sown lentils had lower leaf relative water content (RWC) than normal-sown lentils due to low soil moisture and heat stress delaying seedling emergence (Supplementary Tables 1, 2; Talukdar, 2013), leading to loss of turgor, reduced cell expansion, and suppressed plant growth and development. A decrease in leaf RWC in response to soil moisture stress has been reported in various species (Nayyar and Gupta, 2006). Late-sown lentils had considerably lower chlorophyll a, chlorophyll b, chlorophyll a/b ratio, carotenoids, stomatal conductance, and net photosynthetic rate than normal-sown lentils. The reduced pigment composition and stomatal conductance in late-sown lentils might have reduced the net photosynthetic rate. Higher stomatal conductance in plants increases CO_2_ diffusion into leaves, favoring photosynthesis. Higher net CO_2_ assimilation rates also increase biomass and crop yield (Saraswathi and Paliwal, 2011).

We observed improved pigment composition and photosynthetic rate in treatments sprayed with Fe+Zn+B and Fe+B. Iron is important for chlorophyll biosynthesis, energy transmission, and chloroplast development (Kim and Guerinot, 2007; Brumbarova et al., 2008; Gill and Tuteja, 2011), so the Fe+Zn+B and Fe+B treatments might have improved chlorophyll content in our study. Kumawat et al. (2006) reported that Fe application to pulse crops increased leaf chlorophyll content attributing to Fe being a structural component of chlorophyll. Boron is an important nutrient for carbohydrate and hormone metabolism and translocation. In addition, B is involved in potassium transport into guard cells and thus stomatal opening (Romheld and Marschner, 1991) and photosynthesis. Zinc deficiency inhibits plant development and limits photosynthesis in many plant species (Wang and Jin, 2005). Chandan and Vijay (2018) reported that soil and foliar sprays of Fe and Zn improved chlorophyll content in lentils. Nandan et al. (2018) reported that combining foliar Zn and Fe sprays at the pre-flowering and pod development stages with the recommended fertilizer dose increased chlorophyll content in chickpea. We observed all of the above advantages of these micronutrients in improving various physiological traits in lentils. Zn, Fe, and B also help alleviate abiotic stress (Waraich et al., 2012), as evidenced by the improved leaf RWC, chlorophyll accumulation, and photosynthesis in late-sown lentils with Zn+Fe+B foliar spray.

High temperature decreases soil nutrient uptake, and moisture stress combined with high temperature further enhances the importance of an exogenous nutrient supply. Endogenous Zn, B, and Fe applications might modulate biochemical changes through antioxidant enzymes, as reported by Corrales et al. (2008); Upadhyaya et al. (2013), and Zahoor et al. (2017). Exogenous nutrient application upregulates chlorophyll biosynthesis, delays senescence, and enhances nutrient biofortification, consequently improving the photosynthetic rate and photosynthetic enzymes (Sarwar et al., 2019). Similarly, Zn-mediated regulation of water relations confers heat tolerance by sustaining cell water and osmotic potential under stress conditions. Boron availability enhances stomatal opening, regulating gaseous exchange in stressed environments (Jain et al., 2001; Stavrianakou et al., 2006). The combined application of Zn, Fe, and B spray treatments improved chlorophyll biosynthesis, photosynthetic rate, gaseous exchange regulation, and osmoregulation, helping the late-sown lentil plants to alleviate stress and improve yield.

### Enzyme Activities

Lipid membrane peroxidation by ROS indicates stress-induced damage at the cellular level. An increase in the lipid peroxidation product, MDA, is often used as a plant stress indicator (Jain et al., 2001), generally increasing with temperature extremities. We observed increased MDA content in the control and the treatment sprayed with tap water. High-temperature stress increased MDA content in French beans (Babu and Devraj, 2008) and rice (Pantoja-Benavides et al., 2021). Under normal sowing conditions (November sown), a well-coordinated and responsive antioxidant system balanced oxidative damage to cellular components. Improved RLWC in the treatments sprayed with micronutrients could help maintain membrane stability during stress. Similar to MDA, proline is important for maintaining osmoregulation and accumulating low molecular weight metabolites, such as sugars, organic acids, and amino acids (Gill et al., 2011; Chakhchar et al., 2015). Proline is a major osmoprotectant (Gill et al., 2011) involved in ROS scavenging, protecting cell membranes from oxidative damage. We observed an increased level of proline accumulation in the late-sown lentils and the Zn, Fe, and B treatment. In general, proline concentration increases in response to water stress, resulting in water transfer to plants (Sofo et al., 2004). Different enzymes and metabolites contribute to the antioxidant defense system. We studied the effect of APX and POX antioxidant enzymes that help protect cells by purifying and detoxifying ROS in cells and increasing stress tolerance (Farooq et al., 2009). This enzyme catalyzes the partitioning of O_2_ into either ordinary molecular O_2_ or H_2_O_2_ and is involved in the fine modulation of ROS for signaling (Lin et al., 2010). Late-sown lentils faced stress in both years due to low soil moisture and increased air temperatures, increasing proline, APX, and POX activities, as reported for canola (Rezayian et al., 2018) and chickpea (Khan et al., 2019). The Zn, Fe, and B foliar sprays induced physiological and biochemical responses, such as ROS scavenging, to cope with the stress. The higher proline concentration in water-stressed plants indicates an efficient mechanism for osmotic regulation, stabilization of sub-cellular structures, and cellular adaptation to water stress (Gunes et al., 2008). The decreased MDA buildup in the foliar spray treatments suggests that micronutrients have the potential to alleviate stress by activating the enzymatic response, as seen in cotton (Sarwar et al., 2019) and citrus (Sarwar et al., 2019). Studies have found that heat-tolerant cultivars exhibit higher antioxidant enzyme activity than sensitive wheat (Huseynova, 2012) and chickpea (Khan et al., 2019) cultivars. However, there may be a temperature threshold at which defensive enzyme activity declines, giving ROS the upper hand (Farooq et al., 2009). In our study, the decreased MDA level and increased proline accumulation with micronutrient foliar spray could be attributed to reduced oxidative stress caused by the ROS scavenging response.

Lentil requires low temperatures during vegetative growth and warm temperatures during reproductive growth. The optimal temperature during flowering and pod filling is 30/18°C (max/min); temperatures > 32/20°C (max/min) can drastically reduce lentil seed yield and quality (Saxena, 2009; Malik et al., 2015; Sita et al., 2018). Late-sown lentils had shorter cool and longer hot periods than normal-sown lentils, exposing the crop to heat and moisture stress, particularly, during the reproductive stage. Temperatures varied by 2–3°C between normal- and late-sown lentils during the reproductive stage (pod initiation to maturity) in both years. Furthermore, daily temperatures exceeded 32°C (T_max_) for 3–5 consecutive days for late-sown lentils. The lentil crops were exposed to above-optimal temperatures in both years, especially when sown late. Due to the lack of irrigation, the crops also suffered moisture stress during the terminal stage, particularly, when sown late.

### Seed Nutrients and Protein

Late-sown lentils had significantly lower vital minerals (Fe, Zn, and B) than normal-sown lentils due to heat and/or drought stress. The lower soil moisture might have inhibited mineral translocation into developing seeds, similar to Sehgal et al. (2019) who reported that moisture and heat stress reduced K, Ca, Fe, P, Mn, and Zn contents in lentils. The reduced seed protein content in late-sown lentils indicated impaired protein synthesis due to stress, consistent with drought-stressed beans (Ghanbari et al., 2013a), chickpea (Behboudian et al., 2001), and wheat (Begcy and Walia, 2015). Stress affects seed output by impairing symbiotic nitrogen fixation, increasing oxygen diffusion resistance to root bacteroides, reducing nitrogenase activity, and thus limiting nitrogen availability for protein biosynthesis (Purcell and King, 1996). While seed protein quality largely depends on genotype, environmental stresses can influence it (Triboï et al., 2003). Drought and heat stresses alter protein fractions primarily due to changes in total nitrogen accumulated during seed filling (Triboï et al., 2003). Some legume studies have reported that drought stress reduces mineral accumulation in developing seeds. For example, Fe, Zn, P, and N concentrations decreased in common bean under drought stress and correlated with reduced total protein content (Ghanbari et al., 2013b). In another study, drought stress during pod filling decreased seed nitrogen and protein contents in white, red, and “chitti” bean cultivars (Ghanbari et al., 2013a).

Foliar micronutrient sprays in the field increased N_2_ fixation and nodule mass and thus grain N contents in chickpea, lentils, and lupin (Yanni, 1992). We found that foliar micronutrient sprays also improved grain Zn, Fe, and B contents, even in late-sown lentils. Other studies have reported that foliar micronutrient sprays affected the uptake of N, P, K, S, Zn, and B in rice (Hossain et al., 2001), soybean (Sarker et al., 2002), lentil (Okaz et al., 1994), and mung (Singh and Prasad, 2014) bean.

### Flower and Pod Numbers and Yield

Terminal drought and high temperatures are becoming more common, impacting seed development in cool-season pulses, such as lentils. High temperature has been associated with reduced water availability (Barnabás et al., 2008). In cereals, such as wheat and maize, combined drought and heat stress decreased photosynthesis, stomatal conductance, leaf area, and water use efficiency (Shah and Paulsen, 2003), with similar results reported for chickpea (Awasthi et al., 2014) and lentil (Sehgal et al., 2017, 2019). Cool-season food legumes, such as chickpea (Kaushal et al., 2013) and lentils (Sita et al., 2017a), are adapted to low and mild temperature environments and are thus highly sensitive to heat stress. Sowing date and micronutrient foliar sprays significantly affected lentil yield attributes. The micronutrient foliar sprays helped in reducing the adverse effects of heat and moisture stress to improve seed yield, even under late-sown conditions, particularly, the B+Fe foliar spray, which increased yield by 35–37% yield when compared to the control.

Flower and pod numbers per plant are the most influential yield components, which are closely correlated with seed yield. Zinc, B, and Mo enhanced pod numbers per plant in mung bean (Quddus et al., 2011), chickpea (Valenciano et al., 2011), Zn and B in green gram (Begum, 2014), Zn in French bean (Nasir et al., 2011), and B and Mo in cowpea (Chatterjee and Bandyopadhyay, 2017). Our study contributes to the growing body of literature on the effect of Zn, Fe, and B on lentils. The correlation analysis provides a clear picture of the relationship between improved yield and exogenous application of these micronutrients. The correlation coefficient between a causal factor and the effect (i.e., grain yield) is almost equal to its direct effect.

High temperatures and moisture stress during the reproductive stage reduced lentil yield by decreasing chlorophyll content and impeding photosynthesis. Exogenous application of Zn, Fe, and B improved chlorophyll content, net photosynthetic rate, water relations, and yield in lentil, especially, B+Fe and B+Zn+Fe, which upregulated antioxidant enzyme activities (POX, APX, proline, and MDA) and improved chlorophyll content, net photosynthetic rate, and yield.

## Conclusion

High temperature and moisture stress at the reproductive stage in lentils reduced grain yield by reducing chlorophyll contents and impairing photosynthesis. Exogenous application of micronutrients (Zn, Fe, and B) ameliorated the stress effects. In particular, B+Fe and B+Zn+Fe upregulated antioxidant enzyme activities (POX, APX, proline, and MDA) and improved chlorophyll content, net photosynthetic rate, water relations, and yield. Our study revealed that foliar spray of Zn, Fe, and B ameliorated terminal heat and moisture stress in late-sown lentils. Furthermore, ameliorating high-temperature-induced oxidative stress using foliar micronutrient sprays opens up the possibility of improving plant growth at higher temperatures.

## Data Availability Statement

The original contributions presented in the study are included in the article/Supplementary Material, further inquiries can be directed to the corresponding authors.

## Author Contributions

VV, KS, RN, AP, and SB designed the study. VV and PB conducted the field experiment and data acquisition. VV, SR, LS, and AP conducted the lab analysis. VV, SR, and MC conducted the statistical analyses and wrote the manuscript. VV, AH, and KHS edited the final version of the manuscript. All authors contributed to the article and approved the submitted version.

## Conflict of Interest

The authors declare that the research was conducted in the absence of any commercial or financial relationships that could be construed as a potential conflict of interest.

## Publisher’s Note

All claims expressed in this article are solely those of the authors and do not necessarily represent those of their affiliated organizations, or those of the publisher, the editors and the reviewers. Any product that may be evaluated in this article, or claim that may be made by its manufacturer, is not guaranteed or endorsed by the publisher.
