# Supplementary material for: Foliar Spray of Micronutrients Alleviates Heat and Moisture Stress in Lentil (Lens culinaris Medik) Grown Under Rainfed Field Conditions
Source: Front Plant Sci. 2022 Apr 7;13:847743. doi: 10.3389/fpls.2022.847743 (PMC9021876; doi:10.3389/fpls.2022.847743)
Supplement: Supplementary file 1 [file Data_Sheet_1.docx]

**Table S1.** Canopy temperature and moisture at various crop growth stages (2018–19)

| **Treatment** | **Vegetative stage** | | **Flowering stage** | | **Podding stage** | | **Maturity stage** | |
| --- | --- | --- | --- | --- | --- | --- | --- | --- |
|  | **Temperature (**^o^C) | **Soil moisture (%)** | **Temperature (**^o^C) | **Soil moisture (%)** | **Temperature (**^o^C) | **Soil moisture (%)** | **Temperature (**^o^C) | **Soil moisture (%)** |
| Normal-sown | 22.2 | 27.3 | 24.4 | 24.7 | 25.8 | 21.7 | 29.0 | 16.4 |
| Late-sown | 22.6 | 26.6 | 27.8 | 22.8 | 29.6 | 17.4 | 31.0 | 13.4 |
| Control | 22.3 | 26.3 | 26.3 | 23.7 | 27.9 | 20.1 | 30.2 | 16.0 |
| Tap water | 22.6 | 26.2 | 26.0 | 22.5 | 27.7 | 19.8 | 30.2 | 15.6 |
| Zn@0.5% | 22.5 | 26.7 | 26.3 | 23.3 | 27.5 | 19.7 | 30.1 | 15.0 |
| Fe@0.5% | 22.6 | 27.2 | 26.2 | 23.5 | 27.6 | 19.7 | 30.0 | 14.7 |
| B@0.2% | 22.3 | 26.8 | 26.2 | 23.3 | 27.7 | 18.7 | 30.1 | 14.9 |
| Zn+B | 22.5 | 26.7 | 26.0 | 24.3 | 27.8 | 18.9 | 30.0 | 14.1 |
| Zn+Fe | 22.2 | 27.5 | 26.2 | 24.8 | 28.0 | 20.4 | 29.9 | 14.5 |
| B+Fe | 22.3 | 27.2 | 26.1 | 24.3 | 27.7 | 19.4 | 29.7 | 15.1 |
| Zn+B+Fe | 22.3 | 28.0 | 26.0 | 24.0 | 27.5 | 19.6 | 29.7 | 14.2 |

**Table S2:** Canopy temperature and soil moisture at various crop growth stages (2019–20)

| **Treatment** | **Vegetative stage** | | **Flowering stage** | | **Podding stage** | | **Maturity stage** | |
| --- | --- | --- | --- | --- | --- | --- | --- | --- |
|  | **Temperature (**^o^C) | **Soil Moisture (%)** | **Temperature (**^o^C) | **Soil**  **Moisture (%)** | **Temperature (**^o^C) | **Soil Moisture (%)** | **Temperature (**^o^C) | **Soil Moisture (%)** |
| Normal-sown | 23.3 | 25.1 | 24.4 | 21.7 | 25.8 | 19.7 | 27.9 | 15.5 |
| Late-sown | 26.2 | 23.1 | 28.9 | 17.8 | 30.4 | 15.8 | 33.2 | 12.1 |
| Control | 24.8 | 24.1 | 27.1 | 21.5 | 28.7 | 19.5 | 31.3 | 14.7 |
| Tap water | 24.7 | 24.5 | 26.8 | 20.4 | 28.5 | 18.4 | 31.0 | 14.5 |
| Zn@0.5% | 24.8 | 23.7 | 26.9 | 20.3 | 28.0 | 18.3 | 30.8 | 13.8 |
| Fe@0.5% | 24.7 | 23.8 | 26.8 | 19.9 | 27.9 | 17.9 | 30.8 | 13.9 |
| B@0.2% | 24.8 | 24.3 | 26.9 | 19.8 | 28.1 | 17.8 | 30.4 | 13.6 |
| Zn+B | 24.8 | 24.1 | 26.4 | 18.8 | 28.1 | 16.8 | 30.5 | 13.5 |
| Zn+Fe | 24.9 | 23.7 | 26.5 | 18.9 | 28.4 | 16.9 | 30.1 | 13.1 |
| B+Fe | 24.7 | 24.1 | 26.3 | 18.3 | 27.7 | 16.3 | 29.9 | 12.7 |
| Zn+B+Fe | 24.7 | 23.9 | 26.5 | 17.7 | 27.6 | 15.7 | 30.1 | 12.6 |

**Figure S1:** Differences in pollen germination in Zn + Fe + B (A) treatment and control (B)
